# Effects of Non-surgical Periodontal Therapy on Porphyromonas gingivalis and Filifactor alocis in Chronic Periodontitis Patients With Type 2 Diabetes Mellitus: A Clinico-Microbiological Study

**DOI:** 10.7759/cureus.96524

**Published:** 2025-11-10

**Authors:** Dilip Goswami, Pankaj Dewanjee, Anupam Deka, Rahul Kanungo, Pinku M Talukdar, Monalisha S Borah

**Affiliations:** 1 Periodontics and Oral Implantology, Regional Dental College, Guwahati, Guwahati, IND; 2 Research, Gauhati Medical College and Hospital, Guwahati, IND

**Keywords:** chronic periodontitis, diabetes mellitus, filifactor alocis, non-surgical periodontal therapy, polymerase chain reaction, porphyromonas gingivalis

## Abstract

Background: Periodontitis is a multifactorial and polymicrobial disease associated with microbial dysbiosis. Diabetes mellitus can modify the onset and course of periodontitis and has a bidirectional relationship with chronic periodontitis. *Porphyromonas gingivalis *is considered to be a key pathogen in the progression of periodontitis, and *Filifactor alocis is* a relevant pathobiont in diabetic individuals. The present study was designed to evaluate non-surgical periodontal therapy (NSPT) on the prevalence of *P. gingivalis and F. alocis *in type 2 diabetes mellitus (T2DM) patients with chronic periodontitis.

Objective: To evaluate the effect of NSPT on subgingival detection of *P. gingivalis* and *F. alocis* by polymerase chain reaction (PCR) and on clinical parameters (probing pocket depth (PPD) and clinical attachment level (CAL)) in adults with T2DM and chronic periodontitis at baseline, three months, and six months. The secondary objective is to explore associations between microbial detection and glycated hemoglobin (HbA1c).

Methods: A total of 30 T2DM patients aged between 30 and 70 years with chronic periodontitis (PPD ≥5 mm, CAL ≥3 mm) voluntarily participated in this study. Clinical parameters like PPD and CAL were recorded at baseline, three months, and six months post-NSPT. Plaque samples from subgingival sites were collected from the deepest pockets and analysed for *P. gingivalis and F. alocis* using PCR.

Results: NSPT resulted in improvements that are statistically significant in PPD and CAL measurements (p < 0.05) over the six months. PCR analysis revealed a statistically significant percentage reduction of *P. gingivalis* and *F. alocis* after therapy. The decrease in *P. gingivalis* level was more consistent compared to *F. alocis,* indicating possible microbial resistance to NSPT.

Conclusion: NSPT was associated with improved clinical periodontal status and reduced microbial presence of key periodontal pathogens in T2DM patients. The findings of the present study suggest that NSPT contributes significantly to the improvement of periodontal health in diabetic patients.

## Introduction

Chronic periodontitis is a multifactorial polymicrobial disease widely prevalent among the general population, where the red complex group of bacteria is mostly responsible [1]. Diabetes mellitus and periodontitis have a bidirectional relationship in modifying the onset and progression of periodontal disease and diabetes [2]. Diabetic individuals are found to be more susceptible to periodontal infections due to compromised immune response and altered host inflammatory reactions [3].

*Porphyromonas gingivalis *is a Gram-negative, anaerobic, asaccharolytic bacterium recognized as a keystone pathogen in chronic periodontitis due to its ability to subvert host immunity and promote dysbiosis within the oral microbiome. The bacterium’s ability to synergize with other pathogens and its persistence despite host defences underscore its pivotal role in the pathogenesis of periodontitis [4-6].

*Filifactor alocis *is a fastidious, Gram-positive, obligate anaerobic rod that exhibits trypsin-like enzymatic activity comparable to *P. gingivalis* and *Treponema denticola*. It is well adapted to survive within the periodontal pocket and shares several virulence traits with *Fusobacterium*. First isolated in 1985 from the human gingival crevice as *Fusobacterium alocis*, it was later reclassified into the genus *Filifactor* based on 16S rRNA analysis and renamed *F. alocis*. This organism is capable of adhering to and invading epithelial cells, with these properties being further enhanced in the presence of *P. gingivalis* [6,7].

Non-surgical periodontal therapy (NSPT) encompasses a range of procedures aimed at controlling periodontal infection, promoting tissue healing, and preserving the natural dentition without surgical intervention. It primarily involves scaling and root planing to remove bacterial biofilm and calculus from tooth and root surfaces, thereby reducing inflammation and facilitating gingival reattachment. Effective NSPT also depends on patient education and meticulous plaque control, which are essential for sustaining periodontal health. In most cases, inflammatory periodontal diseases can be effectively managed through these nonsurgical approaches, achieving significant clinical improvement and minimizing the need for surgical procedures.

The present study was designed to evaluate the effect of NSPT on the prevalence of *P. gingivalis* and *F. alocis* in chronic periodontitis patients suffering from type 2 diabetes mellitus (T2DM) by using the polymerase chain reaction (PCR) technique. The primary objective is to assess the impact of NSPT on PCR-based detection of *P. gingivalis* and *F. alocis* and on probing pocket depth (PPD) and clinical attachment level (CAL) at baseline, three months, and six months. The secondary objective is to evaluate correlations between microbial detection and glycated hemoglobin (HbA1c).

## Materials and methods

This quasi-experimental study was conducted at the Department of Periodontics and Oral Implantology, Regional Dental College (RDC), Guwahati, Guwahati, India. A total of 30 research participants with chronic periodontitis suffering from T2DM within the age group between 30 and 70 years were selected from the Outpatient Department of Periodontics, RDC, Guwahati, India. The study duration was for a period of one year, starting from 29/08/2023 to 30/08/2024. The research procedure was briefed to each of the research participants, and informed consent was signed by each of the willing participants. A dental and medical history was recorded, and a thorough intraoral examination was conducted.

Sample size calculation

With an α-set at 0.05, a β-set at 0.95, and an effect size of 0.62, the required sample size was a total of 30 patients.

Ethical permission

This study was approved by the institutional ethics committee (RDC/29/2011/2696) of RDC, Guwahati.

Study sample

Inclusion Criteria

Research participants belonging to the age group between 30 and 70 years were selected, suffering from chronic periodontitis and also from T2DM, with a minimum of 20 permanent teeth having PPD ≥ 5mm or CAL ≥ 3mm. PPD was measured using the University of North Carolina-15 (UNC-15). The glycemic status of participants previously diagnosed with T2DM (having moderately controlled T2DM - 7% to 8% HbA1c levels as per the American College of Physicians) was confirmed by their HbA1c levels, and all the patients selected were in the overweight BMI category. Subgingival plaque samples from the selected test sites were collected with a specific Gracey curette. Clinical parameters such as PPD and CAL were recorded at baseline, three months, and six months post-NSPT. NSPT was performed following plaque sample collection, which primarily included ultrasonic scaling and root planing using Gracey curettes under a sterile environment at both time periods, i.e., baseline and three months follow-up.

Reliability Testing and Calibration

The clinical parameters, PPD and CAL, were examined by a single calibrated examiner to rule out examiner bias prior to sample collection.

Exclusion Criteria

Exclusion criteria included participants who had a history of chronic periodontitis or any other condition and undergone scaling and root planing within the previous six months; those with a history of antibiotic or anti-inflammatory drug use within the past three months; individuals with systemic diseases or conditions other than T2DM; pregnant or lactating women; and smokers or tobacco chewers.

Sample collection

Proper sterilization was observed. Plaque samples were collected from the deepest pocket depth after isolation with sterile cotton rolls in each segment/arch and from two sites each, i.e., mid-buccal and disto-buccal, and were placed in a microcentrifuge tube containing 400 µL phosphate buffer solution (PBS).

Sample transportation

Samples were kept in a biosafety transport icebox and transported to the Multidisciplinary Research Unit (MRU), Guwahati Medical College and Hospital, where it was stored at (-80°C) in a freezer for further genomic analysis by the PCR technique.

Sample processing

DNA Extraction

Collected plaque samples were subjected to DNA isolation using the DNA extraction kit (Qiagen, Germany).

The procedure involved the following steps:

(1) Ethanol (99.9%), molecular grade mix with different buffer solutions, was prepared. (2) PBS mixed with the sample (400 µL) was placed in a vortexer. The tube was centrifuged at 8000 rpm for one minute, followed by incubation at 53°C for 15 minutes in a separate incubator machine. (3) Lysis buffer solution was then added to the solution obtained in step 2. Two separate mixtures were prepared: 300 µL (A) and 600 µL (B). Each was mixed with the sample and centrifuged separately - mix A at 8000 rpm for one minute and mix B at 14,000 rpm for three minutes - and the supernatant was discarded. (4) Wash buffer (500 µL) was mixed with both samples A and B and centrifuged at 8000 rpm for one minute for two rounds, and a dry spin was done at the end. (5) Elution centrifugation is done at 8000 rpm for one minute. The quality analysis of the collected elution fluid (extracted DNA) was done, and it was stored at -20°C until further analysis by the PCR technique.

PCR

PCR was performed using specific primers for DNA amplification of the 16S rDNA gene as described before by Siqueira and Rôças (2004) [8] for *F. alocis* and by Slots et al. (1995) [9] for *P. gingivalis* (Table 1). The PCR amplification was done in a PCR thermocycler machine as per the protocol mentioned in Tables 2-3. The gel was photographed under UV light by a gel documentation system. The bands with molecular sizes of 594 base pairs (bp) for *F. alocis* and 405 bp for *P. gingivalis* were used as a standard for analysing the bacterial strains, with the help of a 100 bp DNA ladder for *F. alocis* and a 50 bp DNA ladder for *P. gingivalis*.

**Table 1:** Primers used for polymerase chain reaction (PCR) PCR primers as described before by Siqueira and Rôças (2004) [8] for *F. alocis* and by Slots et al. (1995) [9] for *P. gingivalis.*

Bacterial Species	Sequence
Filifactor alocis	
Forward	CAGGTGGTTTAACAAGTTAGTGG
Reverse	CTAAGTTGTCCTTAGCTGTCTCG
Porphyromonas gingivalis	
Forward	AGGCAGCTTGCCATACTGCG
Reverse	AGGCAGCTTGCCATACTGCG

**Table 2:** Program for polymerase chain reaction (PCR) amplification of Porphyromonas gingivalis

Temperature	Time	Cycle
95°C	5 min	-
95°C	30 sec	35 cycles
55.2°C	30 sec	
72°C	60 sec	
72°C	10 min	-
4°C	Hold	-

**Table 3:** Program for polymerase chain reaction (PCR) amplification of Filifactor alocis

Temperature	Time	Cycle
95°C	5 min	-
95°C	30 sec	35 cycles
53°C	30 sec	
72°C	60 sec	
72°C	10 min	-
4°C	Hold	-

Statistical analysis

Statistical analysis was performed using GraphPad Prism version 8.0 (GraphPad Software, San Diego, CA, USA). Paired t-test was used for comparisons of continuous variables across different time points. Pearson correlation was performed to analyse associations between different variables. Normality tests were performed and found no significant difference among variables; hence, the data were found to be normally distributed. Gender evaluation was also performed; however, there was no significant difference observed. Hence, the pooled data were presented.

Disposal of the sample

After completion of the PCR technique, the used samples were disposed of according to the standard biomedical waste disposal protocol.

## Results

Demographic characteristics of study participants

**Figure 1:**
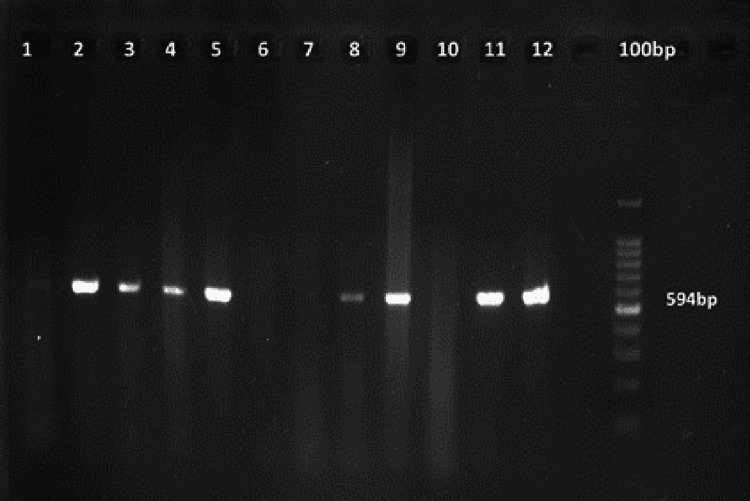
Gel image showing amplification of Filifactor alocis with a product size of 594 bp. bp: base pair

**Figure 2:**
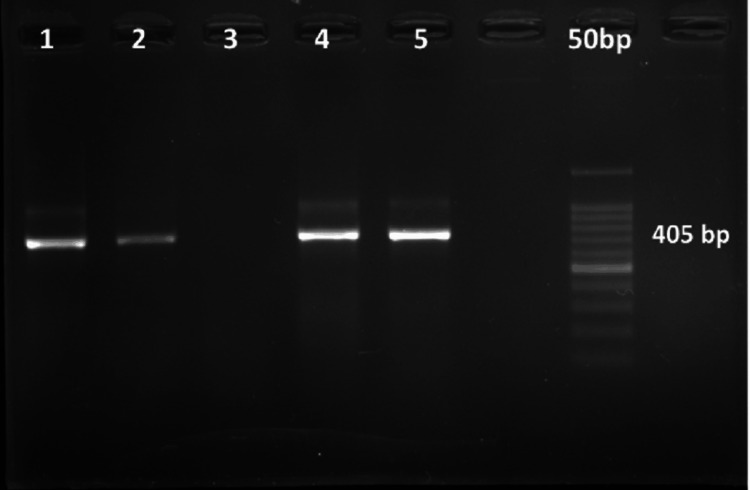
Gel image showing amplification of Porphyromonas gingivalis with a product size of 405 bp bp: base pair

The present study included a total of 30 research participants with a mean age of 49.77 ± 12.57 years. The gender distribution shows a higher representation of males, comprising 66.67% of the sample, while females constituted 33.33%. At baseline, participants had a mean PPD of 7.87 ± 1.2 mm and CAL of 5.87 ± 1.2 mm. At the end of six months of NSPT, there was a significant reduction in both parameters. The mean PPD reduced to 6.6 ± 1.1 mm, and the mean CAL improved to 4.6 ± 1.1 mm, showing positive treatment outcomes. Regarding the microbial findings, *P. gingivalis *was detected in 90% of the participants before treatment. After six months of therapy, bacterial presence was reduced to 26.7%. Similarly, *F. alocis *was found in 86.67% of participants before treatment and was reduced to 30% after therapy. Figure 1 shows the presence of *F. alocis*, with a product size of 594 bp. Figure 2 shows the presence of *P. gingivalis*, with a product size of 405 bp. Findings of the current study indicate the effectiveness of NSPT in lowering *P. gingivalis *​*and F. alocis* (Table 4).

**Table 4:** Demographic details Data are presented as percentages and as mean ± standard deviation (SD). A p-value < 0.05 was considered statistically significant. The total sample size (N) was 30. PPD: probing pocket depth; CAL: clinical attachment level

Clinical Parameters	Mean ± SD (N=30)
Age (years)	49.77±12.57
Gender	
Male (n=20)	66.67%
Female (n=10)	33.33%
PPD (mm) (N=30)	
Pre-treatment	7.87 ±1.2 mm
Post-treatment (six months)	6.6±1.1 mm
CAL (mm) (N=30)	
Pre-treatment	5.87±1.2 mm
Post-treatment (six months)	4.6±1.1 mm
Organisms	
*Porphyromonas gingivalis *(N=30)	
Pre-treatment	90%
Post-treatment (six months)	26.7%
*Filifactor alocis *(N=30)	
Pre-treatment	86.67%
Post-treatment (six months)	30%

At baseline, *F. alocis* exhibited a statistically significant positive correlation with (r = 0.53; p = 0.002), suggesting a potential synergistic interaction within the subgingival microbiota, and demonstrated a moderate positive correlation with HbA1c levels (r = 0.44; p = 0.01), indicating increased prevalence in patients with poor glycemic control, while neither organism correlated significantly with PPD or CAL. At the three month follow-up, the association between *F. alocis* and *P. gingivalis *decreased (r = 0.32; p = 0.09), indicative of a partial microbial response to NSPT, and correlations with clinical parameters remained insignificant; however, *F. alocis* maintained a significant correlation with HbA1c (r = 0.39; p = 0.03), whereas the correlation between *P. gingivalis* and *HbA1c* strengthened (r = 0.61; p = 0.0003), underscoring the influence of hyperglycemia on pathogen persistence. By six months, *F. alocis* exhibited a highly significant association with HbA1c (r = 0.80; p < 0.0001), reinforcing its dependence on metabolic dysregulation, whereas *P. gingivalis* demonstrated moderate positive correlations with both PPD (r = 0.38; p = 0.04) and CAL (r = 0.38; p = 0.04), reflecting its role in progressive tissue destruction, alongside a persistent correlation with HbA1c (r = 0.57; p = 0.001). Clinically, NSPT was associated with improvement in periodontal parameters, with mean PPD decreasing from 7.90 ± 1.2 mm at baseline to 6.60 ± 1.1 mm at six months (p < 0.0001) and CAL improving from 5.87 ± 1.2 mm to 4.60 ± 1.1 mm (p < 0.0001), confirming the efficacy of NSPT in enhancing periodontal status irrespective of microbial persistence. Overall, the findings indicate that while NSPT was found to be associated with positive clinical outcomes, both *F. alocis* and *P. gingivalis* adapt to the host environment, with *F. alocis* demonstrating a stronger dependence on glycemic conditions (Table 5-8).

**Table 5:** Correlation analyses of variables at baseline Pearson correlation analysis of *Filifactor alocis* and *Porphyromonas gingivalis* with clinical variables. Data are presented with 95% confidence intervals (CI). A p-value < 0.05 was considered statistically significant. PPD: probing pocket depth; CAL: clinical attachment level; HbA1c: glycated hemoglobin

	Correlation Coefficient (r)	95% of CI	P-value
Filifactor alocis and Porphyromonas gingivalis	0.53	0.21 to 0.74	0.002
*F. alocis* and PPD (mm)	-0.11	0.25 to 0.45	0.55
*F. alocis* and CAL (mm)	-0.11	0.25 to 0.45	0.55
*F. alocis* and HbA1c (%)	0.44	0.10 to 0.69	0.01
*P. gingivalis* and PPD (mm)	-0.03	0.33 to 0.37	0.89
*P. gingivalis* and CAL (mm)	-0.03	0.33 to 0.37	0.89
*P. gingivalis* and HbA1c (%)	0.41	0.07 to 0.67	0.02

**Table 6:** Correlation analyses of variables at three months follow-up Pearson correlation analysis of *Filifactor alocis* and *Porphyromonas gingivalis* with clinical variables. Data are presented with 95% confidence intervals (CI). A p-value < 0.05 was considered statistically significant. PPD: probing pocket depth; CAL: clinical attachment level; HbA1c: glycated hemoglobin

	Correlation Coefficient (r)	95% of CI	P-value
*Filifactor alocis *and* Porphyromonas gingivalis*	0.32	0.05 to 0.60	0.09
*F. alocis* and PPD (mm)	0.18	0.20 to 0.50	0.35
*F. alocis* and CAL (mm)	0.18	0.20 to 0.50	0.35
*F. alocis* and HbA1c (%)	0.39	0.03 to 0.66	0.03
*P. gingivalis* and PPD (mm)	-0.09	0.28 to 0.44	0.63
*P. gingivalis* and CAL (mm)	-0.09	0.28 to 0.44	0.63
*P. gingivalis* and HbA1c (%)	0.61	0.32 to 0.80	0.0003

**Table 7:** Correlation analyses of variables at six months follow-up Pearson correlation analysis of *Filifactor alocis* and *Porphyromonas gingivalis* with clinical variables. Data are presented with 95% confidence intervals (CI). A p-value < 0.05 was considered statistically significant. PPD: probing pocket depth; CAL: clinical attachment level; HbA1c: glycated hemoglobin

	Correlation Coefficient (r)	95% of CI	P value
*Filifactor alocis *and* Porphyromonas gingivalis*	0.43	0.08 to 0.68	0.02
*F. alocis* and PPD (mm)	-0.05	0.31 to 0.40	0.79
*F. alocis* and CAL (mm)	-0.05	0.31 to 0.40	0.79
*F. alocis* and HbA1c (%)	0.80	0.62 to 0.90	<0.0001
*P. gingivalis* and PPD (mm)	0.38	0.02 to 0.65	0.04
*P. gingivalis* and CAL (mm)	0.38	0.02 to 0.65	0.04
*P. gingivalis* and HbA1c (%)	0.57	0.26 to 0.77	0.001

**Table 8:** Mean differences, paired t-test results, and p-values for PPD, CAL, and HbA1c (%) between baseline, three-month, and six-month follow-up (p < 0.05 = significant) Data are presented as mean ± standard deviation (SD). A p-value < 0.05 was considered statistically significant. PPD: probing pocket depth; CAL: clinical attachment level; HbA1c: glycated hemoglobin

Variable	Baseline (Mean ± SD)	Three Months Follow-Up (Mean ± SD)	Six Months Follow-Up (Mean ± SD)	t-value (Three Months)	t-value (Six Months)	P-value
PPD (mm)	7.90±1.2	7.08±1.2	6.6±1.1	13.38	23.16	<0.0001
CAL (mm)	5.87±1.2	5.05±1.2	4.6±1.1	13.38	22.45	<0.0001
HbA1c (%)	7.87±0.83	7.17±0.68	6.51±0.53	11.13	14.76	<0.0001

## Discussion

This current study was designed to evaluate the clinical and microbiological impacts of NSPT on *P. gingivalis* and *F. alocis* in patients suffering from chronic periodontitis and T2DM using the PCR technique for microbial genomic detection.

Results show a statistically significant improvement in clinical parameters over a six-month period, confirming the therapeutic efficacy of NSPT. The presence of both *P. gingivalis* and *F. alocis* decreased after NSPT.

Although similar studies were reported previously, there is a dearth of data from this particular region. Hence, the present study aims to establish a relationship between *P. gingivalis and F. alocis *in chronic periodontitis patients suffering from T2DM, and there are no known studies done in this region that reported the relationship of these two bacteria with T2DM. Findings of the present study support the polymicrobial synergy and dysbiosis (PSD) model, wherein *P. gingivalis *- a keystone pathogen - plays a central role in restructuring microbial communities to a dysbiotic state [10] and correlates with HbA1c and the organism’s dependency on the metabolic state of the host [7]. *F. alocis*, a fastidious anaerobe, has emerged as an important contributor to the pathogenic biofilm, particularly in diabetic patients, characterized by oxidative stress and immune dysfunction. Significant correlation between *F. alocis* and HbA1c emphasizes its role as a metabolically sensitive pathobiont [11-13].

Understanding microbial dysbiosis within chronic periodontitis patients with T2DM [14] and clinical efficacy of NSPT in reducing pathogenic species like *F. alocis,* having its resistance to oxidative stress, proteolytic capabilities, and its ability to induce apoptosis and modulate immune responses, will help clinicians to properly address the disease [7,14-16]. *F. alocis* and *P. gingivalis*, especially in hyperglycemic conditions, can enhance the pathogenic potential of subgingival biofilm. This microbial shift is further compounded by hyperglycemia-induced oxidative stress and impaired neutrophilic function, which together create an environment conducive to the proliferation of pathobionts like *F. alocis* and *P. gingivalis* [13]. The present study supports that *F. alocis* is elevated in diabetic patients with chronic periodontitis, whereas the abundance of *P. gingivalis* was not significantly different across glycemic conditions, indicating *P. gingivalis'* role may extend beyond dominance to include modulation of community behavior through immune evasive strategies [14,17,18].

Geographical and ethnic variabilities, along with possible primer-binding polymorphisms in *F. alocis*, may account for discrepancies in microbial detection frequencies, warranting deeper metagenomic and meta-transcriptomic exploration for accurate microbial profiling [19]. Patients with better glycemic control exhibited absence of *F. alocis*, reinforcing the well-documented bidirectional relationship between diabetes and periodontal disease, wherein each condition exacerbates the other [2,7,13].

The present study has found that NSPT is effective in reducing *P. gingivalis* and *F. alocis* in diabetic periodontitis patients, and the use of PCR may precisely identify target pathogens and is a valuable tool for monitoring microbial shifts.

However, the major limitation of the study was the small sample size. The study findings may be further validated by a quantitative method used to quantify the bacterial load in conducting a similar study and using a randomized control group in a larger population.

## Conclusions

Observations and findings of the present study highlighted the need for a personalized treatment model for managing periodontitis in patients suffering from diabetes. This should incorporate routine glycemic assessment, molecular diagnostics, and NSPT. Longitudinal research may be designed to explore the roles of* F. alocis *and* P. gingivalis* and to find out the interactions between them and also with other key pathogens. Chairside molecular diagnostic tools could facilitate more personalized and precise treatment planning. This study emphasizes that diabetic patients must be under supportive periodontal care (SPC) with routine professional mechanical plaque removal as a part of SPC, to limit the rate of tooth loss and provide periodontal stability or improvement with controlled glycemic status.

The present study shows that NSPT improved the clinical parameters in the six-month follow-up group by reducing the presence of periodontal pathogens in diabetic patients, although the influence of systemic metabolic factors on microbial dynamics and therapeutic outcomes is to be considered. An integrated, multifactorial approach to treat diabetic patients with periodontitis is needed to achieve optimum periodontal health. Further validation requires quantitative bacterial assessment and a larger randomized control group.

## References

[1] Holt SC, Ebersole JL (2005). Porphyromonas gingivalis, Treponema denticola, and Tannerella forsythia: the "red complex", a prototype polybacterial pathogenic consortium in periodontitis. Periodontol 2000.

[2] Preshaw PM, Alba AL, Herrera D, Jepsen S, Konstantinidis A, Makrilakis K, Taylor R (2012). Periodontitis and diabetes: a two-way relationship. Diabetologia.

[3] Ebersole JL, Holt SC, Hansard R, Novak MJ (2008). Microbiologic and immunologic characteristics of periodontal disease in Hispanic americans with type 2 diabetes. J Periodontol.

[4] Lamont RJ, Hajishengallis G (2015). Polymicrobial synergy and dysbiosis in inflammatory disease. Trends Mol Med.

[5] Olsen I, Lambris JD, Hajishengallis G (2017). Porphyromonas gingivalis disturbs host-commensal homeostasis by changing complement function. J Oral Microbiol.

[6] Mei F, Xie M, Huang X, Long Y, Lu X, Wang X, Chen L (2020). Porphyromonas gingivalis and its systemic impact: current status. Pathogens.

[7] Aruni AW, Roy F, Fletcher HM (2011). Filifactor alocis has virulence attributes that can enhance its persistence under oxidative stress conditions and mediate invasion of epithelial cells by porphyromonas gingivalis. Infect Immun.

[8] Siqueira JF Jr, Rôças IN (2004). Simultaneous detection of Dialister pneumosintes and Filifactor alocis in endodontic infections by 16S rDNA-directed multiplex PCR. J Endod.

[9] Slots J, Ashimoto A, Flynn MJ, Li G, Chen C (1995). Detection of putative periodontal pathogens in subgingival specimens by 16S ribosomal DNA amplification with the polymerase chain reaction. Clin Infect Dis.

[10] Hajishengallis G, Lamont RJ (2012). Beyond the red complex and into more complexity: the polymicrobial synergy and dysbiosis (PSD) model of periodontal disease etiology. Mol Oral Microbiol.

[11] Aruni W, Chioma O, Fletcher HM (2014). Filifactor alocis: the newly discovered kid on the block with special talents. J Dent Res.

[12] Aja E, Mangar M, Fletcher HM, Mishra A (2021). Filifactor alocis: recent insights and advances. J Dent Res.

[13] Taylor GW, Borgnakke WS (2008). Periodontal disease: associations with diabetes, glycemic control and complications. Oral Dis.

[14] Santos Tunes R, Foss-Freitas MC, Nogueira-Filho Gda R (2010). Impact of periodontitis on the diabetes-related inflammatory status. J Can Dent Assoc.

[15] Pandian DS, Victor DJ, Cholan P, Prakash P, Subramanian S, Shankar SP (2023). Comparative analysis of the red-complex organisms and recently identified periodontal pathogens in the subgingival plaque of diabetic and nondiabetic patients with severe chronic periodontitis. J Indian Soc Periodontol.

[16] Shaikh HFM, Oswal PU, Kugaji MS, Katti SS, Bhat KG, Joshi VM (2023). Co-occurrence of Filifactor alocis with red complex bacteria in type 2 diabetes mellitus subjects with and without chronic periodontitis: a pilot study. Int J Transl Med.

[17] Darveau RP, Hajishengallis G, Curtis MA (2012). Porphyromonas gingivalis as a potential community activist for disease. J Dent Res.

[18] Casarin RC, Barbagallo A, Meulman T (2013). Subgingival biodiversity in subjects with uncontrolled type-2 diabetes and chronic periodontitis. J Periodontal Res.

[19] Dabdoub SM, Ganesan SM, Kumar PS (2016). Comparative metagenomics reveals taxonomically idiosyncratic yet functionally congruent communities in periodontitis. Sci Rep.

